# How to Improve Fault Tolerance in Disaster Predictions: A Case Study about Flash Floods Using IoT, ML and Real Data

**DOI:** 10.3390/s18030907

**Published:** 2018-03-19

**Authors:** Gustavo Furquim, Geraldo P. R. Filho, Roozbeh Jalali, Gustavo Pessin, Richard W. Pazzi, Jó Ueyama

**Affiliations:** 1 Federal Institute of Education, Science, and Technology of São Paulo (IFSP), Sao Paulo, CEP: 14801-600, Brazil; 2 Institute of Mathematics and Computer Science (ICMC), University of Sao Paulo (USP), Sao Carlos, São Paulo, CEP: 13566-590, Brazil; geraldop@icmc.usp.br (G.P.R.F.); joueyama@icmc.usp.br (J.U.); 3 University of Ontario Institute of Technology (UOIT), Oshawa, ON L1H 7K4, Canada; roozbeh.jalali@uoit.ca (R.J.); richard.pazzi@uoit.ca (R.W.P.); 4 Polytechnic School, Universidade do Vale do Rio dos Sinos, São Leopoldo, RS, CEP: 93.022-750, Brazil; gpessin@unisinos.br

**Keywords:** wireless sensor networks, internet of things, fault tolerance, disaster forecast, machine learning

## Abstract

The rise in the number and intensity of natural disasters is a serious problem that affects the whole world. The consequences of these disasters are significantly worse when they occur in urban districts because of the casualties and extent of the damage to goods and property that is caused. Until now feasible methods of dealing with this have included the use of wireless sensor networks (WSNs) for data collection and machine-learning (ML) techniques for forecasting natural disasters. However, there have recently been some promising new innovations in technology which have supplemented the task of monitoring the environment and carrying out the forecasting. One of these schemes involves adopting IP-based (Internet Protocol) sensor networks, by using emerging patterns for IoT. In light of this, in this study, an attempt has been made to set out and describe the results achieved by SENDI (System for dEtecting and forecasting Natural Disasters based on IoT). SENDI is a fault-tolerant system based on IoT, ML and WSN for the detection and forecasting of natural disasters and the issuing of alerts. The system was modeled by means of ns-3 and data collected by a real-world WSN installed in the town of São Carlos - Brazil, which carries out the data collection from rivers in the region. The fault-tolerance is embedded in the system by anticipating the risk of communication breakdowns and the destruction of the nodes during disasters. It operates by adding intelligence to the nodes to carry out the data distribution and forecasting, even in extreme situations. A case study is also included for flash flood forecasting and this makes use of the ns-3 SENDI model and data collected by WSN.

## 1. Introduction

In recent years, pollution, uncontrolled development and the consequent damage suffered by the environment in urban areas have led to unprecedented changes in the local climate. Consequently, these changes have increased the number and intensity of natural disasters such as landslides, flash floods and fires, and meant that they have now become a global problem [1]. As recently as 2013, 330 natural disasters were documented and about 96.5 million people were affected, causing an estimated financial loss of US$ 118.6 billion around the world. In the case of flash floods, every year about 102 million people are directly or indirectly affected and this number is constantly increasing [2,3].

Rashid et al. [4] presented in their survey, several applications areas for WSN, where we find energy systems, transport, health-care, gas, air, structures, and urban temperatures monitoring, among others. Despite presenting some works that present forecasts solutions, Rashid et al. [4] described the forces of nature as being brutal and unpredictable, causing material damage and death of millions of people, leaving the lack of energy, food, water, and communication failures for the survivors.

Regarding WSNs application in natural disasters, Rashid et al. [4] listed a series of related works to surveillance, where WSNs were used for detection of transportation and smuggling routes of radioactive material as a long-term solution to nuclear material-based terrorism, and tsunamis detection and response, where WSNs were used for the prediction of tsunamis and floods, using prediction based on web services and neural networks. In addition, WSNs were used as systems to mitigate tsunami and floods effects, most of which, like ours, used water pressure sensors for data collecting and as input for their forecasting models.

In addition, post-disaster actions help to save lives and, in this scenario, it is important to exchange information. Rashid et al. [4] presented works where WSNs were used or modified for this purpose, allowing a more efficient distribution of information among relief disaster teams and hospitals, enabling an improvement in the care of victims. Some of these solutions can be used in other situations besides disaster relief, such as in hostage situations and debris monitoring. In this way, Rashid et al. [4] confirm that the use of WSN is feasible and can achieve good results in the detection and mitigation of damages caused by natural disasters.

In this scenario, wireless sensor networks (WSN) combined with new systems and technologies, such as Internet of Things (IoT), which is a natural part of smart cities and environments, is one of the most promising alternatives to help dealing with the problem of natural disasters in urban areas. It should be noted that in IoT, one of the most important factors is connectivity. It does not matter what the “thing” is but rather how much capacity it has to monitor environments in an architecture that allows the integration of the most wide-ranging objects and an interaction to occur between these objects in a smart way.

One possible course of action is to follow the new trend and adopt sensor networks based on IP by using emerging standards, such as 6LoWPAN/IPv6, which allow the most diverse objects in IoT to be connected with each other [5]. The use of these standards makes communication possible between the WSN, the nodes of a WSN and the Internet, thus enabling the sensor nodes to be viewed as smart objects that form a link between the physical world and online systems [6]. In the management and forecasting of natural disasters, the data collected by means of the sensors can be analyzed together with data available on the Internet such as satellite-based forecasting or types of variables which the sensor nodes are unable to obtain. Moreover, a scenario in which the sensor nodes are connected to the Internet, makes it possible to use technologies such as *Cloud* computing and social networks to help in the forecasting and issue online warnings. In addition, this scenario also allows the sensor nodes to communicate with nearby devices which share the same technology and propagate the information and forecasts.

The need for fault tolerance approaches is another important issue when dealing with natural disasters. This issue is partially addressed in Rehmani et al. [7], where a Cognitive Radio Based Internet Access Framework for Disaster Response for inhospitable environments is presented. In its framework, Rehmani et al. [7] use the term Cognitive for devices that can modify their parameters on-the-fly, being able to adapt to environment changes and, thus, restore the communication between partially damaged networks. Despite addressing only communication, Rehmani et al. [7] make clear the challenges and the need for mechanisms that can recover systems damaged by natural disasters and thus keep their services available to the population.

In the city of São Carlos, Brazil, there is a WSN deployed by the Institute of Mathematical Sciences and Computing (ICMC), University of São Paulo (USP), called REDE (WSN for monitoring urban rivers), the purpose of which is to monitor, analyse the data, detect flash floods, and present online information about rivers in urban areas [8]. The REDE system collects data on the rivers and rainfall in the area and uses two different kinds of technology to communicate, ZigBee and 3G. However, the REDE system does not envisage that this data will be used to forecast flash floods or that this information which is collected in the real world can be integrated with other information available in online environments. Additionally, the REDE system does not have any fault-tolerance mechanism, which is necessary in hazardous environments where the loss of nodes and communication is a common phenomenon.

With regard to this paper, the IoT paradigm allows the REDE system to be extended by introducing the concept of integration between devices and remote services. These services help in the distribution of information and data, as well as in the forecasting and decision-making and act in the real world by sensing and providing information about the monitored environment. This means that the nodes of a WSN (such as the REDE system) can be seen as smart objects that have to meet certain requirements such as the following: (a) diversity in data collection from different types of sensors; (b) the possible use of these data via the Internet and (c) collective intelligence among all/some nodes, based on information about multiple nodes and not only about the situation and data of a single one. Thus, it can be said that the management and forecasting of natural disasters takes place in scenarios where the integration between the real and the online worlds occurs spontaneously [5,6]. How the smart objects concept is employed in our system is explained in Section 3.1 and Section 3.3.

In light of this, the aim of this paper is to propose, design, evaluate and assess the performance of a fault-tolerance system for forecasting and issuing warnings of natural disasters called SENDI (System for dEtecting and forecasting Natural Disasters based on IoT), with a case study about flash flooding. SENDI was implemented by using ns-3, emerging IoT standards and Machine Learning (ML), and by applying real data collected by means of the REDE system. The system is designed for natural disasters in a hostile environment, which is susceptible to failures of communication and loss of nodes (either by physical destruction or a lack of energy). To this end, we also proposed and evaluated a node cooperation mechanism for cluster formation, based on the energy remaining in each node. This mechanism is designed to make better use of the remaining energy in each node, by extending the lifespan of the system and ensuring it is able to keep making forecasts, even in the case of node losses. The forecasting scheme of SENDI relied on embedded models created by means of ML techniques and real data collected by the REDE system within the nodes.

The remainder of this paper is divided as follows: Section 2 describes some related works where IoT and ML are employed in managing and forecasting natural disasters. In Section 3, there is a description of the architecture of SENDI Section 3.1, as well as details about 6LoWPAN and the routing mechanism Section 3.2, the fault-tolerance scheme Section 3.3 and the forecasting models used in the experiments with SENDI. This paper conducts a discussion about the experiments and the results obtained by SENDI in Section 4, and this is followed by the conclusion and suggestions for future work in Section 5.

## 2. Related Works

In Dubey et al. [9] confirms the feasibility of using IoT along with Crowdsourcing for applications aimed at disaster response, together with the challenges this raises. In this paper, the authors describe how during a disaster, such as an earthquake, the users can retain a reduced but usable part of their capacity for communication (despite the destruction of basic facilities), for instance, by using SMS messages. Moreover, as well as the information that the users send, this type of communication contains extra data, such as longitude and latitude coordinates, that can help in the disaster response. A case study was undertaken which involved interviewing selected members of the community. This information was used to identify data sources, both in IoT and crowdsourcing, which could help in the disaster response.

Asta Zelenkauskaite et al. [6] conducted another study on disaster management aided by social networks. His work attempted to design an IoT environment to represent the dynamics of communication and interaction of objects. The model was formed through a process of recognizing and categorizing the properties of smart objects and conducting an analysis of social networks. Since there is a lack of a specialist database for disasters, the model was validated by means of a graph theory combined with the analysis by the CPAN database of the Perl development community.

Deak et al. [10] propose the use of IoT DfPL (Device-free Passive Location), since this enables the users to be located in an environment without the need to have any type of device for disaster management. In their work, the IoT DfPL consists of a WSN DfPL that collects the data. The collected data is sent via the Internet and Analyzed; it thus requires the presence of people in the monitored area during the occurrence of a disaster. Unlike the procedure adopted in this work, at no stage are the data processed in the nodes, even if there is a loss of communication with the base station. Moreover, as in the case of the studies of Dubey [9] and Zelenkauskaite [6], Deak [10] does not make use of a real database with data collected in the targeted environment. Another point which differs from this paper, is the lack of a mechanism for forecasting disasters. With the use of ML for disaster forecasting, precious minutes can be spent on decision-making, which can assist in reducing the loss of materials and lives, even in the event of a node failure.

Arjun et al. [11] set out a mixed architecture that combines WSN with multiple types of sensors and *Cloud* computing for the forecasting of natural disasters. In their study Arjun et al. establish an SaaS (Software as a Service) framework that is integrated with *Cloud*-WSN; this is able to provide virtualization both at the level of the nodes and at the level of the network. The virtualization is delivered with the aim of improving the use of WSN resources. The data are collected by the WSN and sent to the *Cloud* for a climate analysis; these data and results are shared with the authenticated users in the system and warnings are issued if any kind of danger is foreseen. However, in their study Arjun et al. failed to foresee new trends in technology such as IoT either in their architecture or in the use of some kinds of fault-tolerance mechanism if there is a loss of nodes or communication. This is because in their scheme they only work with a simple WSN.

Following the *Cloud* computing idea, Persico et al. [12] show a survey focusing on the performance of different *Cloud* datacenters also presenting an interesting approach to evaluate these performance parameters without being restricted to provider information.

Mitra et al. [13] present a project very similar to the one developed in this paper, which uses WSN, IoT and ML for flood forecasting, but is flood-only and does not take into account fault tolerance. As model for flood prediction, Mitra et al. [13] used RNA and ns-2 for the simulation. As experiments and results, Mitra et al. [13] analyzed the relationship between the number of nodes and the loss of packets and presented, as a measure of performance of the forecast models, the correlation (*R*). Since the coefficient of determination is the square of the correlation, i.e., $R^{2}$, the results obtained by Mitra et al. [13] are very close to those obtained in this article and are presented in the Section 4.2. However, the way the data were selected and used was not described in detail, not allowing to know if the database was restricted to flood situations or not, as is the case of this work.

In an approach that addresses the energy problem, Mostafaei et al. [14,15] present solutions using a different perspective from the one applied in this work, being complementary to the proposed solution, where the machine learning technique Learning Automata is used to address the sleep scheduling and partial coverage problemshelping to save the energy system and prolonging its lifetime.

Table 1 summarizes the most relevant aspects of the cited papers.

## 3. SENDI: System for Detecting and Forecasting Natural Disasters Based on IoT

**Figure 1:**
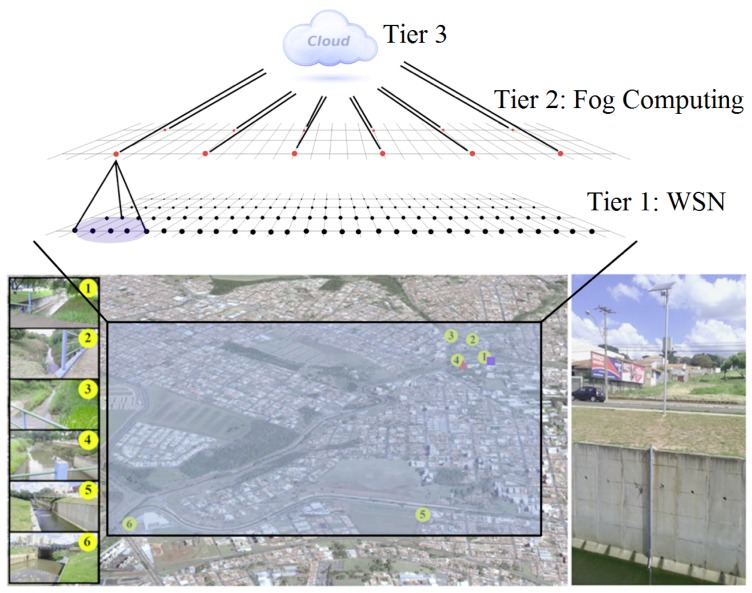
Architecture of the SENDI system viewed from the tier.

This section provides an overview of SENDI, a fault-tolerant system based on IoT and ML for the forecasting and detection of natural disasters. SENDI is formed of 3 tiers, as illustrated in Figure 1. The first tier consists of a WSN, which is responsible for collecting and disseminating data from the monitored environment. The second tier (fog computing) is responsible for concentrating the data in the sensor nodes, as well as carrying out the distribution and processing of the data collected from the first tier. In addition, it should be noted that the second tier is based on the fog computing paradigm, which leaves the computing resources (processing and storage) closer to the end-user so that it can improve the latency of the service. Finally, the third tier consists of *Cloud* where all the data of the system are concentrated and provides processing power and data storage in an abstract form. In this way, the *Cloud* obtains a general view of the monitored environment and is able to combine the data gathered by the sensors with data available on the Internet and make more accurate forecasts, as well as issuing online alerts to the people in the locality.

The conception of this solution arose from the E-Noe project, which was installed in the town of São Carlos and involves flood forecasting and detection and issuing warnings of a possible flood to the residents in the region. For this reason, the main aim of SENDI is to improve the forecasting of natural disasters, while at the same time, providing a fault-tolerant scheme to the system. In the case of this last point, it is essential to ensure that the system is operating, whatever the situation.

The way each tier works is shown in Section 3.1 for a better understanding of SENDI. Following this in Section 3.2, there is an explanation of how the communication took place between the nodes and the dispatch of data to the Internet. After this, the fault-tolerance mechanism is outlined in Section 3.3. Finally, there is an explanation in Section 3.4 of the main features of the data collected by the WSN installed in São Carlos—SP, Brazil and how these data are used to create the model for disaster forecasting (flash floods in this case).

### 3.1. System Architecture

The nodes of Tier 1/WSN which were modeled based on the WSN that can be found in São Carlos—SP, Brazil, form the basis of SENDI. Thus, the nodes do not possess a limited supply of energy, although they have a solar battery charger. In this tier, the basic function of the nodes is data collection. In effect, these nodes have several sensors that are able to pick up different features of the monitored environment and send these data to the Tier 2/fog computing. The nodes of this tier also have audio and visual devices (such as sirens and lights) that are able to warn the local population of the risks of a disaster occurrence.

The nodes of Tier 2/fog computing are in turn responsible for the aggregation of data collected by the sensor nodes and the dispatch of the data to Tier 3/*Cloud*. It should be noted that a node from Tier 2 is only responsible for collecting data from Tier 1 when they are neighbors. Two nodes can be defined as neighbors when they are able to communicate with each other directly without the need for hops. As shown in Figure 1, a node from Tier 2 is responsible for the nodes from Tier 1 that lie within its range of direct communication.

The nodes of Tier 2 are characterized by having a unlimited supply of energy (being that the only difference with respect to Tier 1 nodes) and help reduce the energy consumption of the sensor nodes (Tier 1/WSN) while messages are being sent to Tier 3/*Cloud*. Thus, like the nodes of Tier 1/WSN and for reasons of redundancy, the nodes of Tier 2/fog computing also have audio and visual devices that are able to warn the local population of the risk of a disaster incident.

Finally, Tier 3 is formed by *Cloud*, which provides the necessary resources for the data to be stored, combined with other online information about the monitored environment and processed by means of the forecasting models created via ML. If a risk of disaster is forecast, the *Cloud* also send warnings online and to the nodes of Tier 2/fog computing, which passes on these warnings to the nodes of Tier 1/WSN.

This configuration adds considerable flexibility to SENDI, since it possesses the simplest nodes that only carry out data readings, together with those that support the processing of large amounts of data and combinations of data, while at the same time making forms of energy saving available. This architecture can be easily modified depending on the monitored environment and the resources available in the locality.

### 3.2. Protocol Stack and Routing

**Figure 2:**
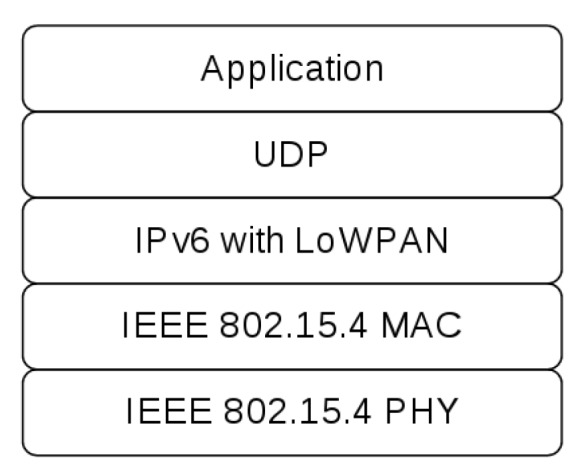
Protocol stack used in the simulation implemented in ns-3.

The protocol stack used by SENDI is shown in Figure 2, where there is an IEEE 802.15.4 standard, that defines the physical layer and the media access control layer of the OSI model for LR-WPAN (Lowdata-Rate Wireless Personal Networks), and Ipv6 in the network layer. The UDP protocol was used on the transport layer because it does not require connection, and is a simple protocol, which generates less overhead in communication; in addition it does not block communication if the target node is not working. On top of the stack, there is the natural disaster monitoring and forecasting application.

As the routing protocol, RFC 6550 defines the RPL (IPv6 Routing Protocol for Low-Power and Lossy Networks) for LLNs (Low-Power and Lossy Networks). LLNs are networks which have restrictions with regard to processing power, energy and/or node memory. The nodes act as routers in these networks and usually only support low data rates, as well as having other specific features such as point-to-multipoint or multipoint-to-point communication standards. Some characteristics of these networks include high number of nodes with hardware and energy limitations, reaching thousands of nodes, all interconnected by lossy links and supporting various traffic patterns [16]. These particular features mean that the routing in these kinds of networks can be distinguished from others and raise their own challenges and solutions [17], as well as clearly describing the properties found in the REDE system.

The RPL protocol was initially based on widely used routing protocols and research prototypes in WSN area, being later extended and re-designed for IPv6. The main implementations of RPL found in the literature are ContikiOS and TinyOS, with TelosB being the most widely used hardware. [16] also comments that, although it already has some years of development and research, the RPL protocol still needs to be tested more deeply in real-world applications and compared with other alternative routing protocol [18].

**Figure 3:**
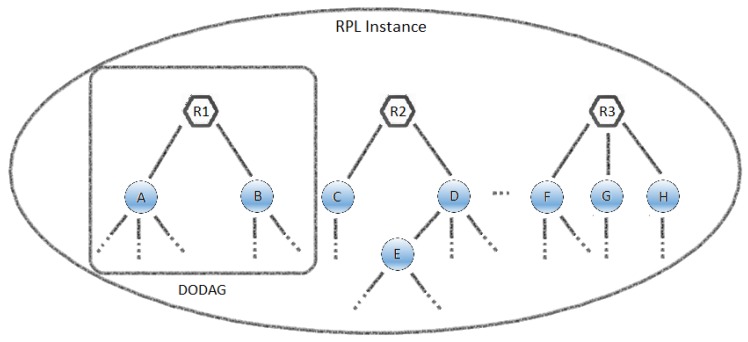
Example of an RPL instance.

Unlike wired networks, where predefined topologies can be found because of the point-to-point, LLNs do not usually have this property which means that they have to discover the links that exist between the nodes and select them carefully. Thus, the RPL arranges the network topology as a set of either a single or several Destination Oriented DAGs (DODAG), that form a Directed Acyclic Graph (DAG), which is a DODAG formed by the sink node (root). Figure 3 shows an example of the configuration of an RPL instance where the R1, R2 and R3 nodes are the roots of their DODAG and are, for example, interlinked with the remaining nodes in the Internet or another network. The nodes that have a direct path to the root, such as nodes A and B, are considered to be Rank 2. All the nodes which are unable to see the root directly but are able to see the Rank 2 nodes are regarded as Rank 3, such as node C from the other DODAG, and so on.

### 3.3. Fault Tolerance Scheme

The fault-tolerance mechanism of SENDI covers failures in the communication and transmission of data between the nodes of any of the tiers and reacts to this failure by ensuring that forecasts of disasters and the issuing of warnings do not cease to be carried out. This kind of failure can be caused either by a lack of energy or the partial or complete destruction of nodes leading to the temporary or permanent loss of their capacity to communicate. To this end, the nodes of Tiers 1 and 2 run a self-organizing algorithm so that they can be reorganized in cases such as this, as well as having embedded disaster forecasting models that are simpler than those processed by *Cloud*. This is because of their energy and processing capacity which allow them to carry out forecasting even if there are communication failures with *Cloud* or the other nodes in the system, although its accuracy is impaired.

In the case of a failure of communication with *Cloud*, the nodes of Tier 2 take on the role of *Cloud*, by storing the data collected by the nodes of Tier 1, distributing these data among themselves and carrying out flood forecasting by employing the forecasting models of natural disasters, as recommended in [19]. In this study, the forecasts carried out by the nodes (similar to the nodes of Tier 2) in a particular region, are sent on to other regions in the monitored environment. In turn, the forecasts that are received, are used to carry out further forecasts in the future and increase the accuracy of the forecasts in the regions of these nodes. Against this background, this paper also extends the work undertaken in [19], which encompasses the work carried out in a forecasting system which includes issuing fault-tolerant IoT alerts.

If failures occur in the nodes belonging to Tier 2, leading to areas being without coverage, where the nodes of Tier 1 remain without a representative in Tier 2 where readings can be sent, the nodes of Tier 1 are rearranged and certain nodes are selected from them to temporarily take the places of the defective nodes in Tier 2. This selection takes place in each round, which is regarded here as a “cycle” of reading and dispatch/distribution of data, forecasting and if necessary, the issuing of warnings. There will be selected as many nodes as needed until all the nodes in Tier 1 have a representative in Tier 2. Basically, the selected nodes play the role of the nodes in Tier 2, by aggregating data from Tier 1 (including their own), sending these data to the *Cloud* and if there is a failure in *Cloud*, carrying out the data distribution and disaster forecasting, in the monitored environment.

**Figure 4:**
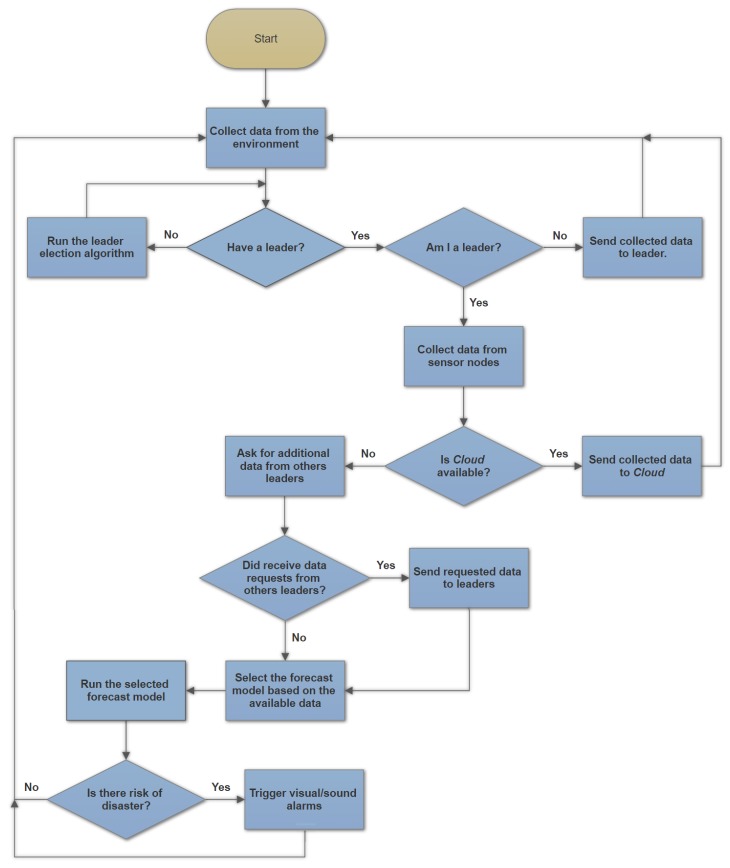
Flowchart representing the rounds of a sensor.

Figure 4 describes the operation of the algorithm for fault tolerance of the system performed by sensor nodes/Tier 1. The sink nodes/Tier 2 perform part of the procedure, except for the leader decision phases, since they are leaders by default.

In serious situations, where there is a loss of communication or failure affecting a large number of nodes, there can be cases where the nodes are completely isolated from others. In this case, as well as being regarded as their own leaders, they are incorporated with the models for simple forecasting that only make use of local data from the nodes themselves. Thus, although they carry out less accurate forecasting and lack the ability to issue warnings online, the nodes can still issue local warnings (such as by means of lights and sirens) and warn the population nearest to the region affected.

The leader election and cluster formation is based on the remaining energy of each node battery, as recommended in this work. It is worth highlighting that these procedures only take place when a possible disaster occurrence is detected or when all the sensing nodes (Tier 1) lose contact with the sink nodes (Tier 2). In each round, all the Tier 1 nodes must have a leader belonging to Tier 2, which groups the collected data and sends them to the *Cloud*. If there is a lack of communication with the *Cloud*, it processes this data and carries out the disaster forecasting. The natural leader of a sensing node in the described architecture is one of the sink nodes of Tier 2. However, if the local sink node fails, the sensing nodes are capable of selecting one of themselves as a new leader and continue the operation of the system. It should also be underlined that unlike the case in other works [20,21,22], the nodes only select their leaders on the basis of their own information. They do not need to know the total amount of energy in the system or any other information about the other nodes in the system.

**Figure 5:**
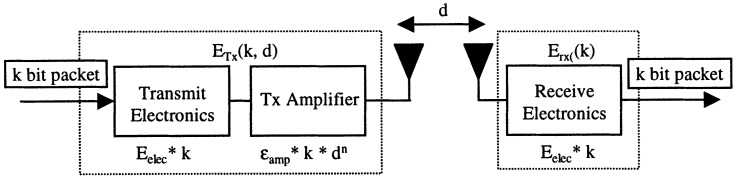
Radio energy dissipation model [23].

With regard to the clustering protocol based on LEACH [23], we employ the simplified model for the radio hardware energy dissipation shown in Figure 5 and use probability to determine whether or not a node will be a leader of a cluster and become part of Tier 2. However, unlike the case of the LEACH protocol, we suppose that: i. initially the nodes may not have the same amount of energy; ii. it is feasible for one node to be the leader on repeated occasions; iii. there is no lower or upper limit for the number of leaders; iv. the process of selection can occur many times in the same round until all the nodes have a leader; v. there is a limit for how many times the selection process can occur for a given node during a round, after this limit has been reached the node is forced to become a leader; and vi. a node can only be accepted as leader of a given node *n* if it belongs to the neighborhood of the node *n*, which reduces the need for hops and for communication with very distant nodes.

According to the radio energy dissipation model, energy consumption caused by the dispatch of a message containing an *l*-bit to a receptor at a distance *d*, for the streaming to have an acceptable Signal-to-Noise Ratio (SNR) is [23]:

(1) $$E_{Tx}\left(l,d\right)=\left\{\begin{array}{c} l\cdot E_{elect}+l\cdot \varepsilon_{fs}\cdot d^{2}\;\;\mathrm{if}\;d<d_{0}\\ l\cdot E_{elect}+l\cdot \varepsilon_{mp}\cdot d^{4}\;\mathrm{if}\;d\geq d_{0} \end{array}\right.$$

where $\varepsilon_{fs}$ and $\varepsilon_{mp}$ depend on the model of the transmitter amplifier for free space (fs) and multipath (mp) and $E_{elect}$ is the consumption of energy of each transmitted bit.

The equation used by the LEACH model to reach the optimal probability of a node and thus become the leader of a cluster, is described in Equation (2) [23]. This probability is used in Equation (4) [23], which defines a threshold. At this moment, given a node *s*, a random number is generated and, if this is smaller than the calculated threshold, the node *s* becomes a leader of a cluster. The details of how to get to this probability can be seen in Heinzelman [23].

(2) $$p_{opt}=\frac{k_{opt}}{n}$$

where $p_{opt}$ is the optimal probability of a node to be a leader, *n* is the number of nodes and $k_{opt}$ is defined as the optimal quantity of clusters and calculated as [23]:

(3) $$k_{opt}=\sqrt{\frac{n}{2\pi }}\frac{2}{0.765}$$

(4) $$T\left(s\right)=\left\{\begin{array}{c} \frac{p_{opt}}{1-p_{opt}\cdot \left(rmod\left(\frac{1}{p_{opt}}\right)\right)}\;\mathrm{if}\;s\in G\\ 0\;\;\;\;\;\mathrm{otherwise} \end{array}\right.$$

As previously stated, the LEACH does not allow a node to be a leader repeatedly. For a node to be a leader again, it must be in set *G* of Equation (4) [23]. This set only contains the nodes which had not been leaders in the most recent (r∗mod(n/k)) rounds, where *k* is the number of expected leaders and *r* is the number of rounds in which the node *s* was not determined as the leader.

In this work, the threshold defined by LEACH was altered to take account of the remaining energy in the node. Thus, given a node *s*, its new threshold is defined as:

(5) $$T^{\prime }\left(s\right)=T\left(s\right)\cdot \alpha +\frac{E}{E_{f}}\cdot \left(1-\alpha \right)$$

where *E* is the current battery power of the node, $E_{f}$ is the maximum capacity of the battery (allowing for differences in the batteries among the nodes), α is the weight given to each part of the equation, where value 1 only uses the LEACH altered/implemented in the simulation, and value 0 only uses the information about energy. After the threshold has been calculated, the procedure continues in the same way, with *s* generating a random number and defining whether or not it is a leader based on a comparison between this value and the threshold. The number of rounds *r* in *s* is reset every time *s* becomes a leader. Apart from these differences, there is no longer a limitation of (r∗mod(n/k)) rounds, thus any node can be a leader regardless of how many times the other nodes have been leaders or the number of rounds (allowing a node with a high battery level to become the leader on repeated occasions). In a same round, this process of selection can take place several times if the first election is not enough for all the nodes to have a defined leader. The maximum number of elections in a round can be stipulated and if a node does not have a leader defined after reach this limit, the node itself becomes his own leader regardless its battery level.

The Algorithm 1 illustrates the operation for the leader election in each node. After each election attempt, if the node was not set as a leader, it waits a random time letting the other nodes in its neighborhood run the same algorithm. In case of a new leader does not appear, the election algorithm continues until it reaches the stipulated maximum limit, when the node is forced to become a leader.

**Algorithm 1** Leader Election Algorithm.  1:$\mathrm{Kopt}\leftarrow \sqrt{\frac{n}{2\times \pi }}\times \frac{2}{0.765}$  2:$\mathrm{Popt}\leftarrow \frac{Kopt}{n}$  3:$\mathrm{ThresholdLEACH}\leftarrow \frac{Popt}{1-Popt\times (rmod\left(\frac{1}{Popt}\right))}$  4:MaxNumberElection←GetMaxNumberOfElections()  5:ElectionCounter←0  6:**while** “Node without a leader” **do**  7: $\mathrm{ThresholdEnergy}\leftarrow \frac{GetCurrentBatteryPowerLevel()}{GetFullBatteryPowerLevel()}$  8: Threshold←ThresholdLEACH×α+ThresholdEnergy×(1−α)  9: RandomNumber←random(0,1)10: ElectionCounter←ElectionCounter+111: **if** RandomNumber<Threshold or ElectionCounter>MaxNumberElection then12:  SetNodeAsLeader()13: **else**14:  wait(random(0,1))15: **end if**16:**end while**

### 3.4. Flash Flood Forecasting with the Use of ML

**Figure 6:**
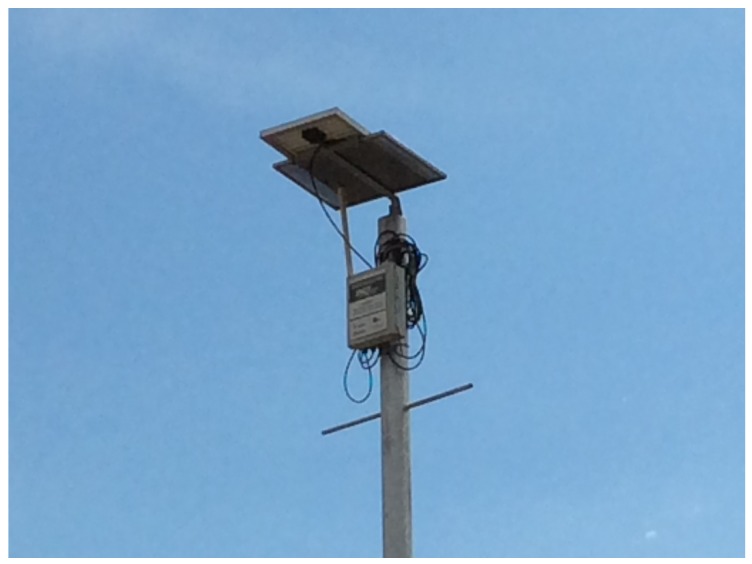
Example of a REDE node.

This section describes the characteristics of the data used in the case study on flood forecasting and how the simple forecast models have been created. It should be stressed that these simple models are only used if there is a failure in communication with the *Cloud* and the Tier 2 nodes, since this makes use of data from just one node. The WSN REDE is composed of 7 nodes, as shown in Figure 6, which are installed in the rivers of the city of São Carlos, Brazil. All the nodes use sensors which read the water pressure on the river bed and convert this value into the depth of the river, measured in centimeters, except in the case of the nodes where the sensor is a rain gauge that measures the volume of rain in the area. Another node that stands out from the others is the node installed in the mall of the town, which is also equipped with a camera that takes pictures of the monitored region. The data used for this study were collected between October 2013 and October 2014 from the node in the mall. The readings of the river level were taken at 1-min intervals and the area was the part of the town which experienced most frequent flooding.

**Figure 7:**
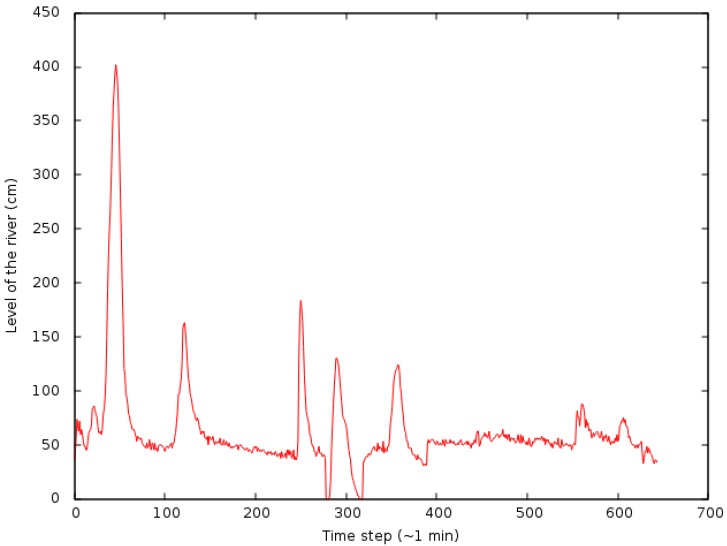
River level dataset used to generate the forecast models.

The readings selected to generate the models were only those which represent the times when the river levels rose or declined. These readings were separate from the others and formed the dataset shown in Figure 7. The models were generated by this dataset and through the implementation of the MLP (Multilayer Perceptron) that is present in the WEKA software [24].

The parameters of the MLP for training were: learning rate = 0.3, 10-fold cross-validation as a test analysis and 1 hidden layer with 3 neurons. The inputs in the MLP are composed of the average of 5 readings. Two forecast models were generated for each node and, given the current time as *t*, in one of the models the output of MLP is the forecast of the average river level from t+τ to t+4+τ (5 minutos in the future—used for generate red alerts), where the τ parameter is detailed in the Section 4.2. The output of the other model is the forecast of the average of the river level from t+5+τ to t+9+τ (5 minutos in the future—used for generate red alerts). These parameters were defined on the basis of previous research, as in [19,25].

## 4. Experiments and Results

SENDI was modeled and tested with the aid of the ns-3 [26], which is an Internet-oriented discrete-event network simulator licensed by GNU GPLv2 and the 6LoWPAN module. The implementation of this module was based on RFCs4944 and 6282 and, despite not bringing to bear the entire set of standards described for 6LoWPAN, it serves as a basis for other functionalities, as it is an official module integrated to ns-3 since ns-3.19 version.

Unfortunately, a routing protocol for the lr-wpan module of ns-3 is not available yet, including the RPL (Section 3.2). Thus, a simplified routing protocol was implemented in ns-3 in order to better assess the performance of the propose scheme. This protocol is based on the position of each node, where a packet with a non-neighbor node as its destination is passed along to another neighbor node which has a potion that is closer to that of the destination node. This path is followed until the packet reaches the destination node. Thus, the nodes also have to know their neighbors’ position and the position of the nodes that will be sent a package. In addition, the position of the destination node is embedded in the sent packet. This routing protocol will not be described in detail as it is beyond the scope of this study.

As the lr-wpan module of ns-3 does not yet have metrics for energy, the simple radio energy dissipation model described in Section 3.3 was adopted in the simulation to determine the energy consumption during the transmission and reception of data. This model was adopted because it is widely used in the literature allowing the comparison of the results obtained. The simple battery model available in ns-3 was also adopted to analyze the battery power consumption. Table 2 shows the parameters adopted in the original LEACH paper and in the simulation, as well as the amount of energy used by the leader of the cluster for each node that belongs to it. This value was adopted because of its impact on the quantity of stored energy in the leader node. Thus, it makes it really necessary to have a mechanism to control the energy, as well as to allow the use of several ML techniques that can be combined or even to handle different kinds of data collected by distinct sensors such as, for instance, images, river level, rainfall, etc.

### 4.1. Fault Tolerance and Clustering

During the initial experiments, communication was interrupted with the sink nodes and the *Cloud*, which made the use of the mechanism for selecting the leader necessary. The experiments consisted of the repetition of rounds which continued until the total amount of energy in the system was consumed and all the nodes had become inactive. The model was employed 33 times for each value of α adopted (Equation (5)) and the averages of the number of nodes working on each round are shown in Table 3. The last row of the table refers to the scalability testing of the system, using the best α value obtained (α = 0.5) but running the simulation with 500 nodes instead of 100.

As can be seen, due to the characteristics of the simulation, the consumed energy in each round remains almost constant and only has variations with regard to how the consumption is shared between the nodes. Thus, all the performances had the same starting-point when the nodes began to be inactive due to lack of energy, being the only difference the number of nodes that remain active in each round. Hence, α values = 0.8, 0.9 and especially 0.5 distributed the charge better over the nodes, thus allowing more nodes to remain working in the following rounds. This is an important factor as if there are more active nodes, a greater amount of data can be collected and a larger region included in the monitored area. As a result, it is possible to perform more accurate forecasts or even alert a larger number of people.

### 4.2. Evaluation of the Flash Flood Forecasting Method

In the case of the experiments related to flood forecasting, data were used from the sensor displayed in Figure 7. Furthermore, assuming the current time is *t* for instance, cases of floods were included to forecast when the average river level from t+1 to t+5 (5 min in advance) or the average from t+6 to t+10 (5 min in advance after t+5) was higher than 2 m. The average from t+1 to t+5 was considered to be enough to cause alerts with a high risk of flooding (classified as red alerts). The average from t+6 to t+10 was considered to be enough to trigger alerts (classified as yellow), when there is the risk of flooding. The average value of 2 m was defined for the location because of the local features of the environment. It was noticed that in a 5-min interval there is the possibility that the river will rise by 3 m, which represents the occurrence of a flood in the region. This means that, if the river level reaches 2 m, there might be a flood in the following 5 min. It is worth remembering that in this critical case only local data of the cluster is taken into account. In an analysis using data from other nodes, as in the case of the system fully operational, the accuracy of the forecasts can be refined.

To determine the input and output parameters of the forecast models, created by the MLP, we used the procedure described in [25]. In this approach, the river level time series (Figure 7) is analysed using Chaos Theory, more specifically the Takens’ Immersion Theorem [27], where the time series is unfolded to better extract its behavior and facilitating its analysis. However, unlike [25], we used only the peaks of the river level to make this study and thus, extending the previous work.

According to Takens [27], a time series $x_{0},x_{1},\ldots ,x_{n-1}$ can be reconstructed in a multidimensional space $x_{n}\left(m,\tau \right)=\left(x_{n},x_{n+\tau },\ldots ,x_{n+(m-1)\tau }\right)$, also called time-delay coordinate space, where *m* is the embedded dimension and τ represents the time delay (or separation dimension). To estimate the separation dimension (τ), Fraser and Swinney [28] propose the use of the Auto-Mutual Information technique (AMI), where the first minimum is the main candidate for τ.

**Figure 8:**
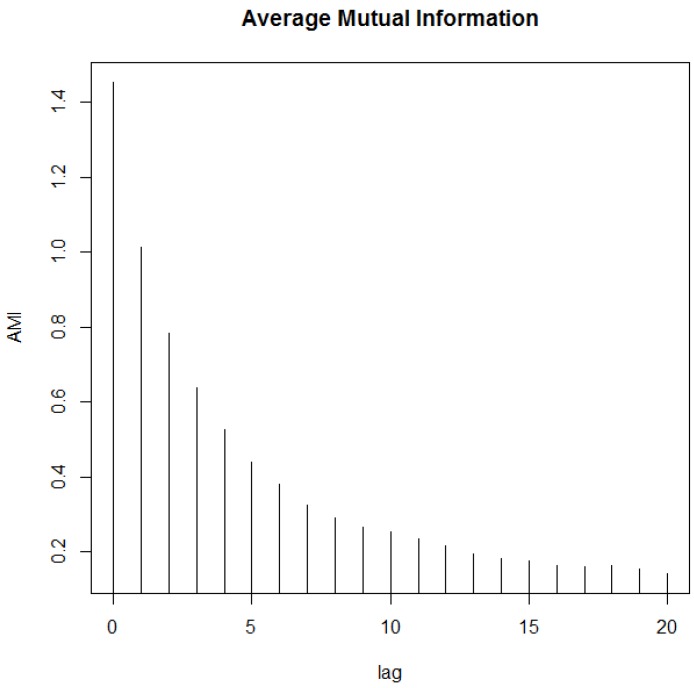
Auto-Mutual Information for the river level.

After estimating the separation dimension, the embedded dimension (*m*) can be estimated using the False Nearest Neighbors method (FNN), as proposed by Kennel [29], where *m* is selected when the fraction of false neighbors is equal to zero. Applying this procedure, the AMI for the river level time series is shown in Figure 8.

**Figure 9:**
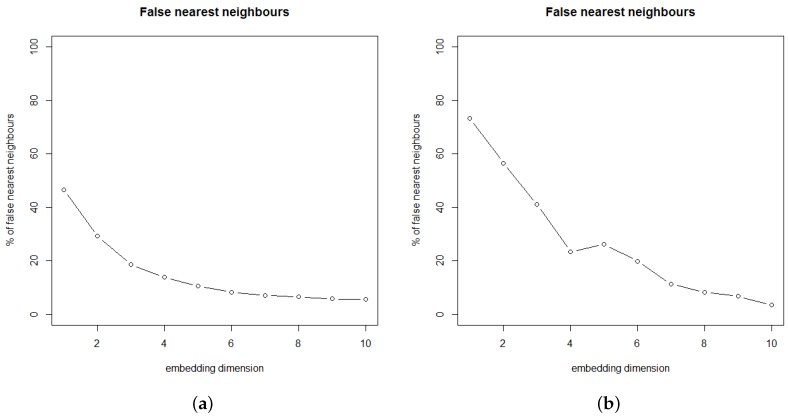
Percentage of false neighbors for the river level, considering (**a**) τ = 1 and (**b**) τ = 17.

The first minimum obtained makes τ=17 but when AMI slightly reduces as the separation dimension increases, another good option for the separation dimension is τ=1, as described in [25,29]. These two values for τ were used to estimate the embedded dimension (*m*) as shown in Figure 9.

Real-world time series, as the ones obtained in natural environments, may contain noises making it difficult to obtain a value for *m*, where the fraction of false neighbors is equal to zero. In such cases, we can select the number of dimensions in which the fraction of false neighbors becomes less than 30%, as presented in [25,30]. Thus, we selected m=3 when τ=1 and m=4 when τ=17 for the unfolding process. Table 4 shows the mean absolute error, root mean squared error and the coefficient of determination ($R^{2}$) obtained by the MLP after unfold the river level time series using these parameters.

At this point, we can compare the results obtained in this work and results of Mitra et al. [13], where seven different flood prediction models were used, each combining information from different sensors. In their work, Mitra et al. [13] used the MLP to create the models, having a hidden layer containing 12 neurons, but it is not described in detail how they selected the amount of input data for each model. These results are presented in Table 5, where the correlation coefficient ($R^{2}$) is not presented in the original work but is calculated from the correlation (*R*), by reasons for comparison.

As we can see, based on the $R^{2}$, the results obtained and presented by the approach of this paper surpass five of the models presented in Mitra et al. [13], losing only to the last two models, which use data of more than one sensor.

**Figure 10:**
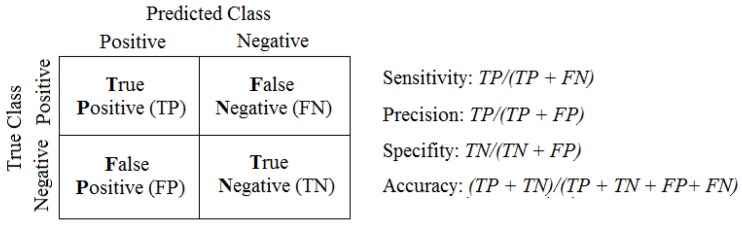
Performance measurements calculated by the confusion matrix [31].

As shown in Table 4, the best results were achieved using the model created by using τ=1 and m=4, therefore the average river level from t−9 to t−5 and from t−4 to *t* are used to forecast the average river level from t+1 to t+5, assuming current time as *t*. In this way, we continue using this model for the next experiments with SENDI and evaluate the results obtained based on the following measures of performance: sensitivity, precision, specificity and accuracy. These measures are calculated from a confusion matrix presented in Figure 10. Also, using m=4 determines that every Tier 1 node must store at least the last 30 consecutive own readings. This is necessary to feed the local forecast model. In this way, tier 1 nodes must be able to store at least 30 floats values and be able to execute a simple MLP with 1 hidden layer with 3 neurons.

The simulation was performed again, although this time with attention being paid to the results of the forecasts. A forecasting model created using the training data was embedded in one of the sensors and fed with readings about the river level during the simulation. The confusion matrices of the embedded model, including averages ranging from t+1 to t+5 (red alert) and from t+6 to t+10 (yellow alert), are shown in Table 6 and Table 7.

**Figure 11:**
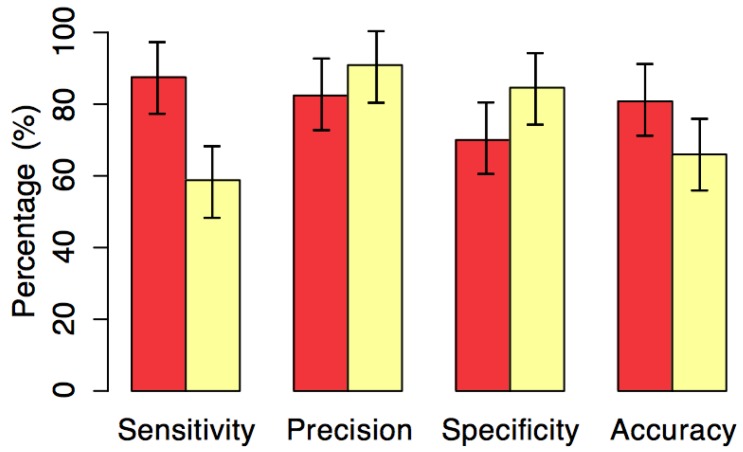
Evaluation of the results obtained by SENDI.

According to [32], these measurements have inherent features which are the total sensitivity of the samples where the results are in a really positive category (i.e., positive truths). Precision is obtained from the total number of examples that can be classified as positive but are not always so; in other words, they are negative truths because specificity is the opposite of sensitivity. This involved classifying as negative, the examples which really are negative (false positives) and the accuracy will make it possible to analyze what method is needed to provide an appropriate classification of a piece of equipment. Following this method, the assessment of the predictive capacity of the SENDI system is shown in Figure 11 with a confidence of 95%, by using the t-student test.

As noted in Table 6 and Table 7 and Figure 11, the accuracy of both alerts exceeds 65% and the red alert has a higher percentage of accuracy (80%) than the yellow alert (66%). Similarly, the percentage of false positives of the red alert is well below that of the yellow alert. However, an in-depth analysis was conducted of the percentage of false negatives, where there is the occurrence of flooding that has not been forecasted by the system. In this respect, the red alert far outweighs the yellow alert and has a much lower failure rate. Although the situation described involves a scenario where there are several failures in SENDI and in a better scenario the distribution and combination of data from other sensors can be used to improve the results of both alerts, as seen in [19].

## 5. Conclusions and Future Works

In this paper, we have examined a fault-tolerant system for detecting and forecasting natural disasters based on IoT and ML called SENDI (System for dEtecting and forecasting Natural Disasters). The system was modeled with the aid of the ns-3 simulator and complied with the IoT standards available in the simulator. The MLP models were designed by Weka and the data collected by means of a WSN deployed in a real environment. A clustering system was adopted to address the failures of the system, such as loss of nodes or communication, giving SENDI a fault-tolerance capacity. In this system, nodes are rearranged on the basis of their remaining energy to keep the ability to continue doing forecasts. This clustering system was analyzed by means of experiments and achieved good results both improving the utilization of the total energy of the system and giving SENDI a fault-tolerant mechanism.

A case study on flood forecasting was also included and used to analyze SENDI. In this case study, we used data collected from urban rivers by means of WSN to create forecasting models. These models were embedded in the SENDI nodes and used to generate alerts in a scenario where faults had previously occurred in various parts of the system. As a result, SENDI was able to make predictions with a high degree of accuracy even in an unfavorable scenario. In future work we intend to expand SENDI by analyzing the characteristics already described for the system (such as the forecasting models present in *Cloud*) and assigning new features such as the combination of readings from multiple nodes, even in the event of system failures. In addition, a distributed approach could be adopted to improve the accuracy of the forecasts, where multiple nodes share forecasts with each other. This could assist in distributing the workload and making combined forecasts with greater accuracy, as intended for the Tier 2. Moreover, a limitation of SENDI is the need for a large number of nodes, therefore, a study about the required number of nodes to create a robust and not easily degrade version of the system still needs to be done. Also, additional experiments using data present in other studies, as in Mitra et al. [13], as well as other leader election algorithms, for instance, TEEN (Threshold-sensitive Energy Efficient sensor Network), other machine learning techniques and enhanced tuning of the forecast models parameters should be performed to achieve a fairer and deeper comparison.

## Figures and Tables

**Table 1:** Papers summary.

	Synthesis
Dubey et al. [9]	Use IoT and Crowdsourcing to disaster response, also presenting a case study
Asta Zelenkauskaite et al. [6]	Present a IoT environment assisted by social network to help disaster management
Deak et al. [10]	Propose the use of IoT DfPL to enable users to be located and disaster management
Arjun et al. [11]	Combine WSN and *Cloud* computing to forecast natural disasters
Mitra et al. [13]	Present an WSN, IoT and ML approach for flood forecast
Mostafaei et al. [14,15]	Propose, present and discuss the results of alternative approaches to address the energy
	limitation problem
Persico et al. [12]	Present a survey and an approach focusing on the evaluation of *Cloud* datacenters performance

**Table 2:** Simulation parameters [23].

$E_{elect}$	$5*10^{-8}$ nJ/bit
$\varepsilon_{fs}$	$1*10^{-11}$
$\varepsilon_{mp}$	$1.3*10^{-15}$
*E*	15,000 J
$E_{f}$	241,920 J
*d*	87.7 m
*n*	100
Voltage and Amperage (idle)	0.5 A / 5.0 V
Leader energy consumption	50 J

**Table 3:** Number of active nodes per round.

α	Round 18	Round 19	Round 20
0.0	100	5	0
0.1	100	7	0
0.2	100	4	0
0.3	100	6	0
0.4	100	8	0
0.5	100	12	0
0.6	100	6	0
0.7	100	6	0
0.8	100	10	0
0.9	100	10	0
1	100	7	0
0.5 (500 modes)	500	464	36

**Table 4:** Multilayer Perceptron: Mean squared error.

	MAE	RMSE	$R^{2}$
τ=1 and m=3	12.9704	22.9193	0.6813
τ=17 and m=4	31.9467	48.6781	0.0024

**Table 5:** MLP correlation and coefficient of determination [13].

Model	Description	*R*	$R^{2}$
A	Only rain as input (RN)	0.5745	0.3301
B	Only moisture as input (HM)	0.2521	0.0636
C	Only water flow as input (WF)	0.8512	0.7245
D	RN + HM	0.9713	0.9434
E	HM + WF	0.8914	0.7946
F	RN + WF	0.9891	0.9783
G	RN + HM + WF	0.9912	0.9825

**Table 6:** Percentage of hits and misses in the forecasts (red alert).

		Predicted Class	
		Positive	Negative
**True Class**	**Positive**	28	4
	**Negative**	6	14

**Table 7:** Percentage of hits and misses in the forecasts (yellow alert).

		Predicted Class	
		Positive	Negative
**True Class**	**Positive**	20	14
	**Negative**	2	11
